# Prognostic Value and Clinicopathology Significance of MicroRNA-200c Expression in Cancer: A Meta-Analysis

**DOI:** 10.1371/journal.pone.0128642

**Published:** 2015-06-02

**Authors:** Jianchun Wu, Zhihong Fang, Jing Xu, Weikang Zhu, Yan Li, Yongchun Yu

**Affiliations:** 1 Department of Oncology, Shanghai Municipal Hospital of Traditional Chinese Medicine, Shanghai University of Traditional Chinese Medicine, Shanghai, China; 2 Shanghai Municipal Hospital of Traditional Chinese Medicine, Shanghai University of Traditional Chinese Medicine, Shanghai, China; University of North Carolina School of Medicine, UNITED STATES

## Abstract

MiR-200c has been shown to be related to cancer formation and progression. However, the prognostic and clinicopathologic significance of miR-200c expression in cancer remain inconclusive. We carried out this systematic review and meta-analysis to investigate the prognostic value of miR-200c expression in cancer. Pooled hazard ratios (HRs) of miR-200c for overall survival (OS) and progression-free survival (PFS) were calculated to measure the effective value of miR-200c expression on prognosis. The association between miR-200c expression and clinical significance was measured by odds ratios (ORs). Twenty-three studies were included in our meta-analysis. We found that miR-200c was not significantly correlated with OS (HR = 1.41, 95%Cl: 0.95-2.10; *P* = 0.09) and PFS (HR = 1.12, 95%Cl: 0.68-1.84; *P* = 0.67) in cancer. In our subgroup analysis, higher expression of miR-200c was significantly associated with poor OS in blood (HR = 2.10, 95%CI: 1.52-2.90, *P*<0.00001). Moreover, in clinicopathology analysis, miR-200c expression in blood was significantly associated with TNM stage, lymph node metastasis and distant metastasis. MiR-200c may have the potential to become a new blood biomarker to monitor cancer prognosis and progression.

## Introduction

Cancer is a class of diseases involving out-of-control cell growth. A total of 1,660,290 new cancer cases and 580,350 cancer deaths are projected to occur in the United States in 2013[1]. Efforts have been made to find new biomarkers to predict the survival and provide information for clinical treatment. Recently, lots of biomarkers have been evaluated in various cancers, such as caveolin in breast cancer[2], C-reactive in urological cancer[3], MMP-9 in esophageal squamous cell carcinoma[4], CD147 in ovarian cancer[5] and S100A4 in colorectal cancer[6]. However, simple and reliable predictors that can be widely used in clinical practice are not currently available. Therefore, there is still a great need to find novel and suitable biomarkers to predict treatment response and outcome of cancer patients.

The miRNA-200 family containing five members (miR-200a, miR-200b, miR-200c, miR-429, and miR-141), is commonly involved in human health and disease. The five members of miR-200 are found in two clusters. MiR-200a, miR-200b, and miR-429 are located on chromosome 1p36 and miR-200c and miR-141 are on chromosome 12p13[7]. MiR-200c is highly enriched in epithelial tissues[8]. MiR-200c is believed to repress the expression of ZEB1 and ZEB2 and has a direct influence on epithelial-to-mesenchymal transition (EMT)[9]. In EMT, the miR-200c is lowly expressed, while ZEB1 and ZEB2 are highly expressed. ZEB1 and ZEB2 bind to the promoter region of CDH1, blocking the synthesis of E-cadherin, which is necessary for intercellular adhesion[10].

In the recent time, the prognostic value and clinicopathology significance of several miRNAs in cancer has been analyzed by meta-analysis[11–14], and some studies have investigated the tumor suppressive function of miR-200 family members in breast[15], colorectal[16] and ovarian[17] cancers. Moreover, some studies have shown that expression of miR-200c is associated with patient prognosis and clinicopathology significance[17–38]. Therefore, it is necessary and timely to perform a meta-analysis to evaluate the prognostic value and clinicopathology significance of miR-200c expression in patients with cancer.

To investigate whether the miR-200c expression could serve as a prognostic or clinical biomarker for cancer, we performed the systematic review and meta-analysis by extracting summary statistics of the published literature for survival endpoints.

## Materials and Methods

### Literature search

The PRISMA statement (S1 PRISMA Checklist) was followed in our meta-analysis. We comprehensively searched Cochrane Library, OVID, PubMed, Web of Science databases and China National Knowledge Infrastructure (CNKI) until March 10, 2015. The key words in searching was “miR-200 OR miR-200c OR miR200 OR miR200c” AND “tumor OR neoplasm OR cancer OR carcinoma.” Moreover, we also checked review articles and references of relevant studies to supplement our search. Oncomine and The Cancer Genome Atlas (TCGA) were searched to make our data sufficient enough. J. Wu and Z. Fang respectively searched the database to get the original data.

### Eligibility criteria

If the following conditions were met, the studies were included in this meta-analysis (a) proven prognosis or clinicopathology significance of the miR-200c expression in cancer; (b) analyzed the correlation of miR-200c with survival outcomes or clinical parameters; (c) registered more than 30 patients. The titles and abstracts were read by two researchers (J. Wu and Z. Fang) independently, and irrelevant studies would be excluded; then our review team would check the full-text and get the essential data.

### Data extraction

Two reviewers (J. Wu and Z. Fang) independently extracted the following data using a form: first author, year of publication, study location, cancer type, number of patients, distribution of age and gender, tumor stage, method of miR-200c detection, cut-off level to consider miR-200c as highly expressed and sample types. Multivariate analysis would be selected because it takes into consideration confounding factors and thus is more accurate [39]. If HRs were not reported in the article, we used Engauge Digitizer version 4.1 (free software down-loaded from http://sourceforge.net) to read the Kaplane-Meier survival curves to get the HRs and their 95% CIs. Two independent authors (J. Wu and Z. Fang) checked the curves in order to reduce reading variability. If there were insufficient data, controversies, or any other uncertainties in an article which might be related to our meta-analysis, we asked corresponding authors for additional information.

### Statistical analysis

We measured the effective value of miR-200c expression on prognosis by hazard ratios (HRs) and corresponding 95% confidence intervals (CIs). An HR greater than 1 indicated poor prognosis in patients with high expression of miR-200c. The association between miR-200c expression and clinical significance was measured by odds ratios (ORs) and 95% CIs. And P values<0.05 were considered to denote statistical significance. Two independent authors (J. Wu and Z. Fang) checked the curves to reduce reading variability. The heterogeneity among the studies was measured using Cochran's Q test and Higgins I-squared statistic. Random-effects models were utilized in order to avoid the influence of heterogeneity. These statistical analyses were conducted using Review Manager Version 5.1 software (http://ims.cochrane.org/revman). The publication bias was examined by R (http://cran.r-project.org/bin/windows/base). To validate the association between miR-200c expression in blood and TNM stage, 1000 re-sampling groups were produced by the bootstrap re-sampling procedure[40,41]. The re-sampling statistic program was shown in S1 Excel File. Furthermore, a randomly generated result was displayed in S2 Excel File. The types of each 692 samples were introduced in S1 Excel File. No.1 was for high expression of miR-200c and high tumor stage; 2 was for low expression of miR-200c and high tumor stage; 3 was for high expression of miR-200c and low tumor stage; 4 was for low expression of miR-200c and low tumor stage. In a randomly generated result, 5000 samples would be produced by bootstrap re-sampling procedure in each re-sampling group and the ORs were automatically calculated. The user could get new random data via pressing F9. The overall ORs containing all samples and the ORs distribution of each re-sample group were displayed in S2 Excel File.

## Results

### Characteristics of identified studies

**Fig 1:**
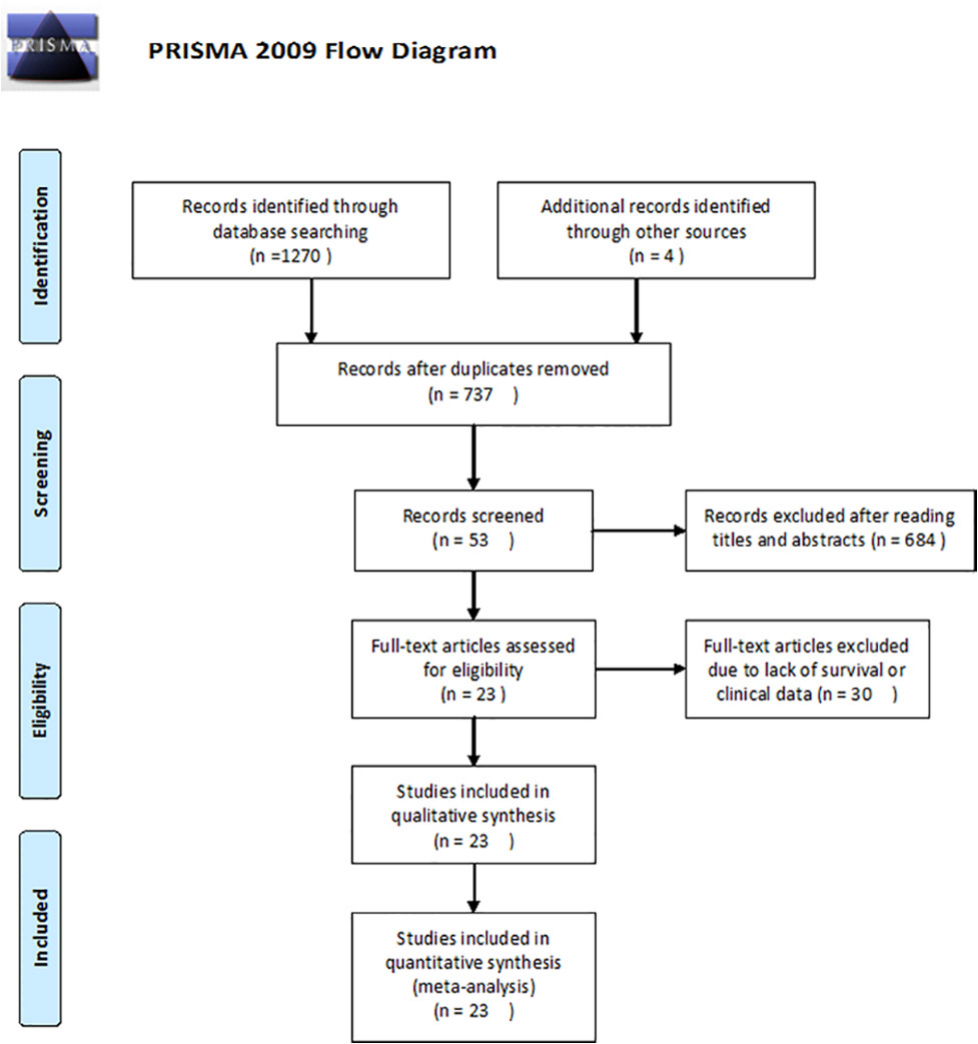
Flow diagram summarizing the selection of eligible studies.

After the primary literature search in database, 245 studies were found in PubMed, 335 studies were found in Web of Science, 119 studies were found in OVID and 571 studies were found in CNKI. Moreover, there were 4 studies found when the authors examined the reference list of the review article. After duplicated studies were excluded, 737 studies were remained. Investigators carefully read the title and abstract then excluded 684 irrelevant studies. Next, the full-texts of the rest articles were reviewed in detail. There were twenty-three studies included in our meta-analysis at last[17–38] (Fig 1). The baseline characteristics of eligible studies were summarized in S1 Table. The included studies were published between 2010 and 2014. There were 2777 participants from Italy, China, Japan, Norway, India, Korea, Poland, Spain and Germany. The malignant carcinomas involved in this review included ovarian cancer, esophageal cancer, lung cancer, colorectal cancer, gastric cancer, breast cancer, pancreatic cancer, endometrioid endometrial cancer and non-metastatic renal cell cancer. All studies use qRT-PCR to detect miR-200c expression.

### Meta-analysis

**Fig 2:**
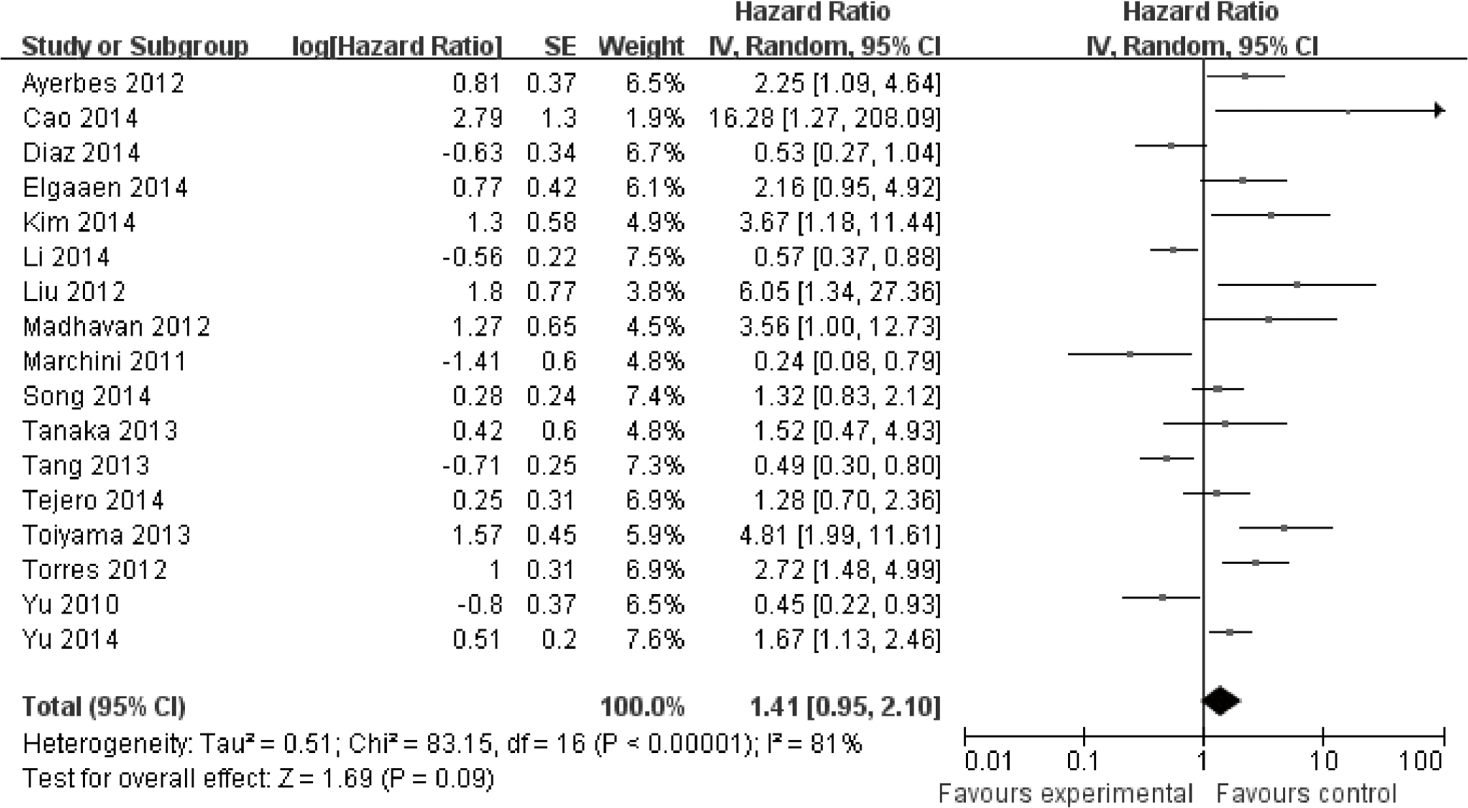
Meta-analysis evaluating miR-200c expression and overall survival (OS) in cancer patients.

Overall, seventeen studies reported data on miR-200c expression and OS in cancer. The combined analysis of the 17 studies showed that expression of miR-200c was not significantly correlated with OS in cancer(HR = 1.41, 95%Cl: 0.95–2.10; *P* = 0.09) (Fig 2). For studies evaluating PFS, expression of miR-200c was not correlated with PFS in cancer (HR = 1.12, 95%Cl: 0.68–1.84; *P* = 0.67) (S1 Fig).

### Subgroup analysis

**Fig 3:**
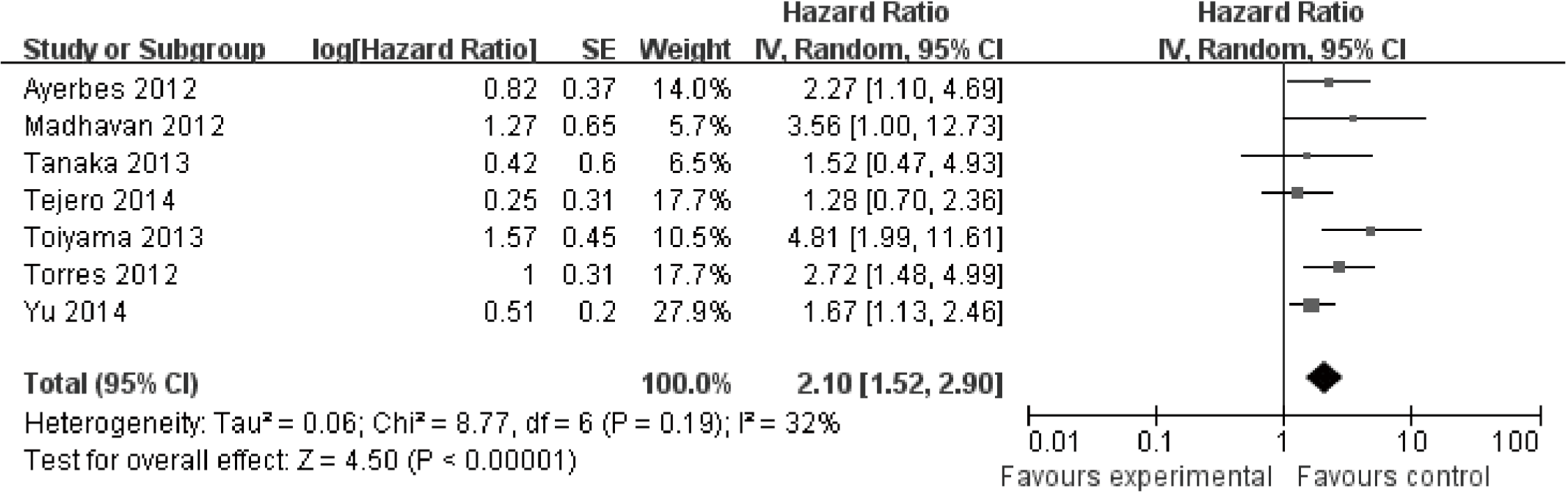
Meta-analysis evaluating miR-200c expression and overall survival (OS) for blood samples.

To get further insights, we performed subgroup analysis with respect to ethnicity and sample type to evaluate miR-200c prognostic value in cancer. As shown in Table 1, expression of miR-200c was not significantly correlated with OS in Caucasians (HR = 1.37, 95%Cl: 0.74–2.53; *P* = 0.32) (S2 Fig) and Asians (HR = 1.46, 95%Cl: 0.85–2.52; *P* = 0.17) (S3 Fig). Expression of miR-200c was also not significantly associated with OS in tissue (HR = 0.99, 95%Cl: 0.59–1.67; *P* = 0.97) (S4 Fig). However, in blood, miR-200c expression was significantly associated with OS (HR = 2.10, 95%CI: 1.52–2.90, *P*<0.00001) (Fig 3).

**Table 1:** A summary of hazard ratios (HRs) for the overall and subgroup analyses of miR-200c expression of cancer patients.

	No. of studies	Pooled HR	95%CI	P-value	Heterogeneity	
					I^2^(%)	P-value
OS						
Overall	17	1.41	0.95–2.10	0.09	81	<0.00001
Caucasians	7	1.37	0.74–2.53	0.32	77	0.0002
Asians	10	1.46	0.85–2.52	0.17	83	<0.00001
Blood	7	2.10	1.52–2.90	<0.00001	32	0.19
Tissue	10	0.99	0.59–1.67	0.97	79	<0.0001
Gastric cancer	3	1.10	0.47–2.57	0.82	86	0.0008
Ovarian cancer	3	1.60	0.23–11.43	0.64	85	0.002
Lung cancer	4	1.67	0.65–4.31	0.29	83	0.0006
PFS						
Overall	8	1.12	0.68–1.84	0.67	80	<0.0001
Blood	3	2.27	1.65–3.12	<0.00001	0	0.78
Tissue	5	0.75	0.51–1.11	0.15	42	0.14

OS, overall survival; PFS, progression-free survival

### Sensitivity analysis

Sensitivity analysis was carried out through omitting one study each time and calculating the pooled HRs again. As shown in S2–S7 Tables, the stability of the entire study was not influenced by one individual study.

### Publication bias

Publication bias was evaluated by Begg’s funnel plot and Egger’s test. The result was displayed in Table 2. Begg’s funnel plot and Egger’s test didn’t suggest any evidence of publication bias.

**Table 2:** Begg’s funnel plot and Egger’s test of publication bias on the relationships between miR-200c and prognostic value in cancer.

	Begg's funnel plot		Egger's test	
	Z test for plot asymmetry	P value	t value	P value
OS				
Overall	1.56	0.120	1.71	0.106
Caucasians	0.25	0.803	-0.23	0.827
Asians	0.54	0.592	1.64	0.140
Blood	1.00	0.319	1.47	0.193
Tissue	1.07	0.283	1.61	0.146
PFS				
Overall	0.25	0.803	-0.27	0.796
Blood	0.64	0.526	1.58	0.159
Tissue	1.02	0.307	1.53	0.181

OS, overall survival; PFS, progression-free survival.

### Clinicopathology analysis

**Fig 4:**
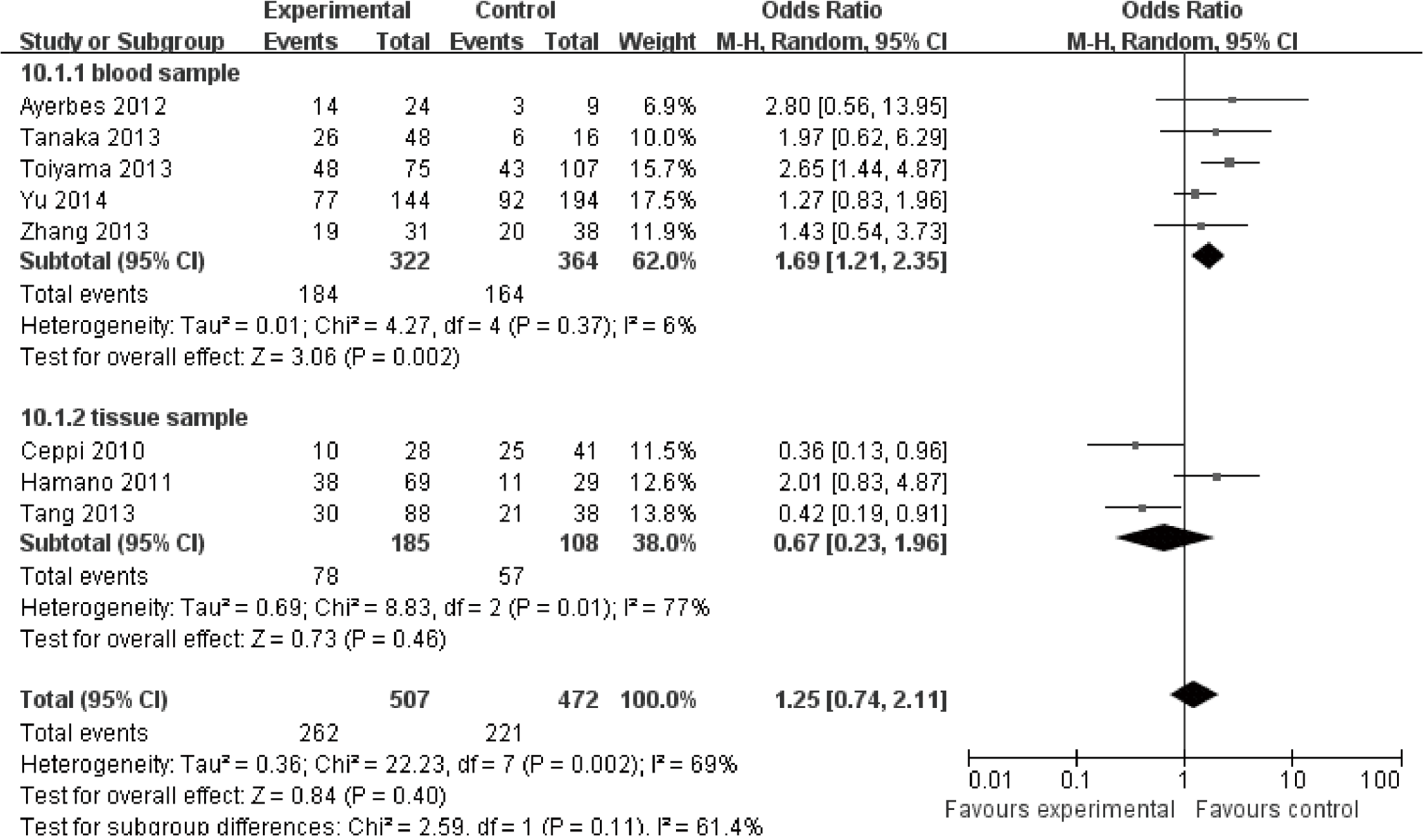
Meta-analysis evaluating miR-200c expression and lymph node metastasis in cancer patients.

**Fig 5:**
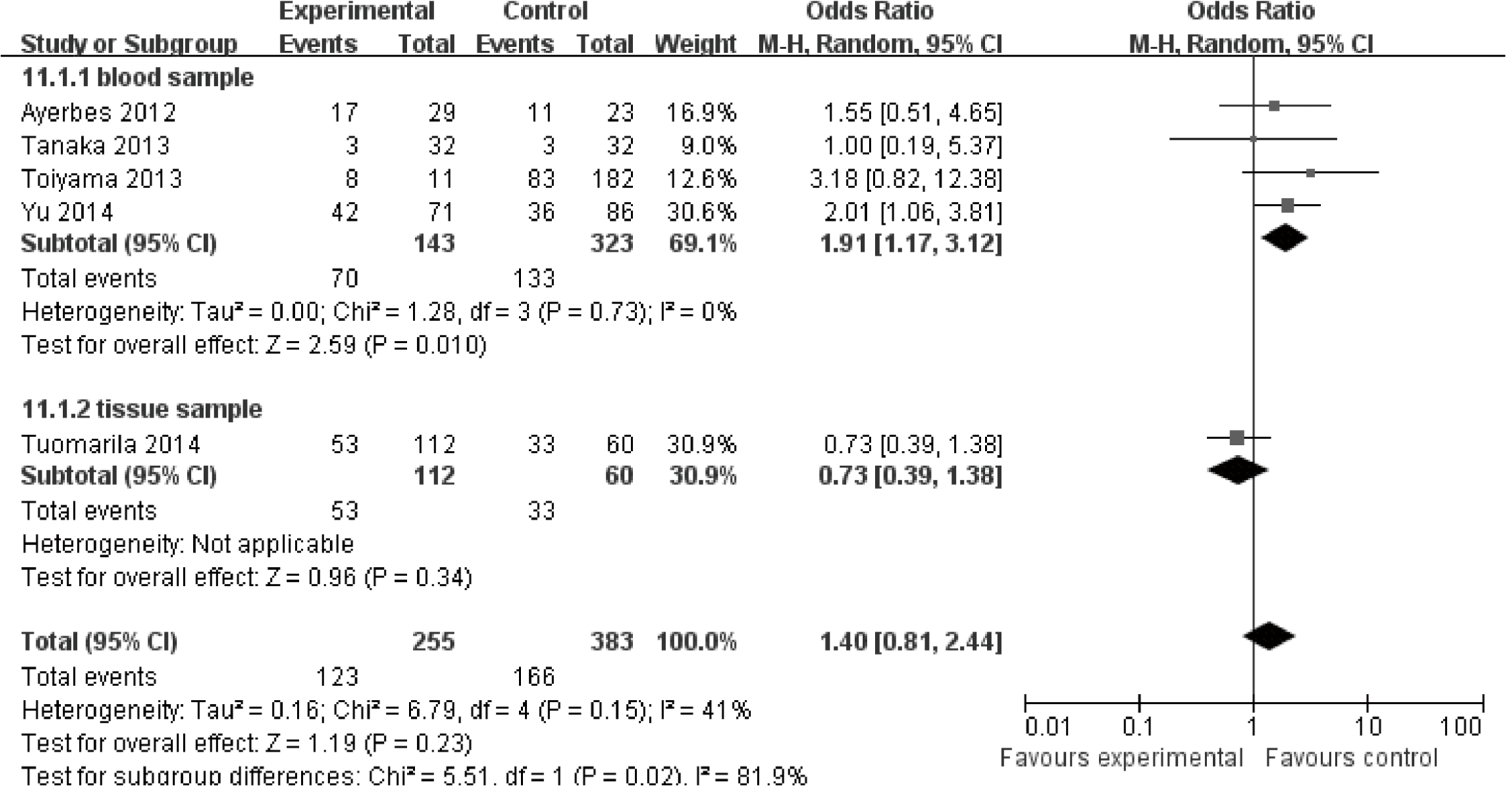
Meta-analysis evaluating miR-200c expression and distant metastasis in cancer patients.

Eleven studies were enrolled in the clinicopathology analysis. Higher expression of miR-200c was significantly associated with higher TNM stage (HR = 1.74, 95%CI: 1.06–2.86, *P* = 0.03) (S5 Fig). No significant association was revealed between miR-200c expression and tumor differentiation (HR = 0.93, 95%CI: 0.61–1.42, *P* = 0.72) (S6 Fig) lymph node metastasis (HR = 1.25, 95%CI: 0.74–2.11, *P* = 0.40) (Fig 4) as well as distant metastasis (HR = 1.40, 95%CI: 0.81–2.44, *P* = 0.23) (Fig 5).

**Fig 6:**
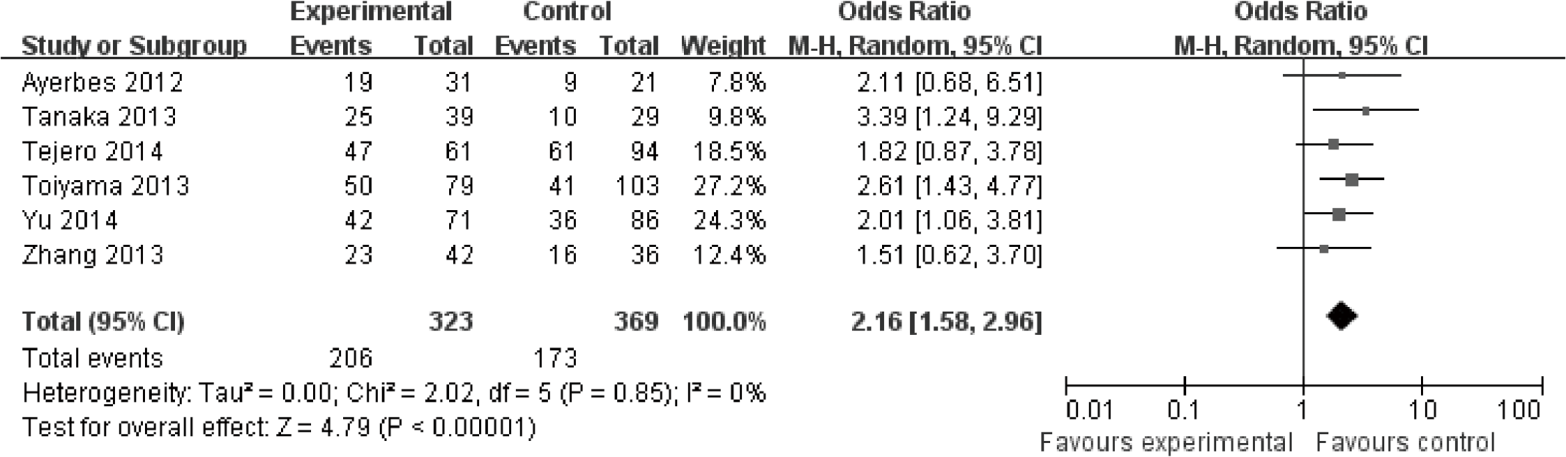
Meta-analysis evaluating miR-200c expression and TNM stage for blood samples.

In blood, higher expression of miR-200c was significantly associated with higher tumor stage (HR = 2.16, 95%CI: 1.58–2.96, *P*<0.00001) (Fig 6). Moreover, higher expression of miR-200c was significantly associated with more lymph node metastasis (HR = 1.69, 95%CI: 1.21–2.35) (Fig 4)and more distant metastasis (HR = 1.91, 95%CI: 1.17–3.12) (Fig 5).

### Re-sampling statistics

Bootstrap re-sampling procedures were applied to investigate the association between miR-200c expression and TNM stage in blood. One randomly generated results were displayed in S2 Excel File. Odds ratios were mostly distributed between 1.72 and 2.27 among 1000 re-sampling groups. The odds ratio was 1.97 when evaluating 5000000 samples (95%CI: 1.97–1.98, *P*<0.00001) (S7 Fig).

## Discussion

MiR-200c is believed to repress epithelial mesenchymal transition (EMT) and tumor metastasis. For instance, increased miR-200c expression leads to a reversal of EMT in bladder cancer[42]. MiR-200c can also inhibit cancer stem cell self-renewal and attenuate differentiation[43].MiR-200c were confirmed to be downregulated in human breast cancer stem cells as well as in normal human and murine mammary stem/progenitor cells. Moreover, miR-200c has a modulatory function in cell division and apoptosis[44]. High expression of miR-200c in cancer was reported in various type of cancer including ovarian cancer, glioma, non-small cell lung cancer (NSCLC), colorectal cancer, gastric cancer, breast cancer, pancreatic cancer and non-metastatic renal cell cancer. In this study, we aimed to explore the association between miR-200c expression and cancer prognosis and clinicopathology.

In our meta-analysis, expression of miR-200c was not significantly correlated with OS in cancer(HR = 1.41, 95%Cl: 0.95–2.10; *P* = 0.09). For studies evaluating PFS, expression of miR-200c was not correlated with PFS in cancer (HR = 1.12, 95%Cl: 0.68–1.84; *P* = 0.67). However, in our subgroup analysis, we found that high expression of miR-200c was significantly associated with poor OS in blood (HR = 2.10, 95%CI: 1.52–2.90, *P*<0.00001). And significant association between miR-200c expression and TNM stage, lymph node metastasis as well as distant metastasis in blood was observed. Re-sampling statistics were used to get robust and replicable results and confirm that higher expression of miR-200c was significantly associated with higher TNM stage. In our sensitivity analysis, we found that the stability of the entire study was not influenced by one individual study. No publication bias was observed. So we can conclude that miR-200c may serve as a blood biomarker for cancer.

MiRNAs are detectable in blood and circulating miRNAs have the potential to be new biomarkers in patients with cancer. The usefulness of miRNA expression as a blood biomarker has been explored in breast cancer[45], esophageal squamous cell cancer[46], hepatocellular carcinoma[47] and non-small cell lung cancer[48]. However, the mechanism how miRNAs in blood affected invasion and metastasis of tumor was not fully uncovered. Increased levels of expression of epithelial-specific miRNAs in blood, including miR-200c, might indicate the circulation of tumor cells which might be closely associated with tumor invasion and metastasis[23].

However, there were still some limitations in our meta-analysis. Firstly, only articles in English were included in our meta-analysis. Strictly, some eligible studies published in other language would be missed. Secondly, some HRs were calculated according to the data extracted from the survival curve, several tiny errors might be brought. Thirdly, cut-off values were different among these studies, we could not set up a baseline referring to miR-200c high expression and inconsistency might be observed. Fourthly, studies included in our meta-analysis were not sufficient, which led to the relative insufficiency of studies in subgroup analyses. The prognostic value of miR-200c in certain tumor type and tissue sample was not fully elucidated because of the insufficient studies. More studies were needed to evaluate the association between miR-200c expression and the prognosis of certain type of cancer.

In summary, our study demonstrated that miR-200c expression was not significantly associated with cancer prognosis. However, miR-200c expression in blood was significantly associated with prognosis, TNM stage, lymph node metastasis and distant metastasis. More studies are needed to confirm the association between miR-200c expression and cancer.

## Supporting Information

S1 PRISMA Checklist Preferred Reporting Items for Systematic Reviews and Meta-Analyses: The PRISMA Statement. (DOC)

S1 Excel File Re-sampling program evaluating miR-200c expression and TNM stage. (XLS)

S2 Excel File Re-sampling results of miR-200c expression and TNM stage. (XLS)

S1 Fig Meta-analysis evaluating miR-200 expression and progression-free survival (PFS) in cancer patients. (TIF)

S2 Fig Meta-analysis evaluating miR-200 expression and overall survival (OS) in Caucasians. (TIF)

S3 Fig Meta-analysis evaluating miR-200 expression and overall survival (OS) in Asians. (TIF)

S4 Fig Meta-analysis evaluating miR-200 expression and overall survival (OS) in Asians. (TIF)

S5 Fig Meta-analysis evaluating miR-200 expression and overall survival (OS) for tumor stage. (TIF)

S6 Fig Meta-analysis evaluating miR-200 expression and overall survival (OS) for tumor differentiation. (TIF)

S7 Fig Meta-analysis evaluating miR-200 expression and overall survival (OS) for tumor stage. (TIF)

S1 Table Baseline characteristics of studies included in the meta-analysis. (DOC)

S2 Table The influence of individual study on the pooled estimate (OR) for overall survival. (DOCX)

S3 Table The influence of individual study on the pooled estimate (OR) for progression-free survival. (DOCX)

S4 Table The influence of individual study on the pooled estimate (OR) for overall survival in Caucasians. (DOCX)

S5 Table The influence of individual study on the pooled estimate (OR) for overall survival in Asians. (DOCX)

S6 Table The influence of individual study on the pooled estimate (OR) for overall survival in tissue samples. (DOCX)

S7 Table The influence of individual study on the pooled estimate (OR) for overall survival in blood samples. (DOCX)

## References

[1] Kuschel B, Auranen A, McBride S, Novik KL, Antoniou A, Lipscombe JM, et al (2002) Variants in DNA double-strand break repair genes and breast cancer susceptibility. Hum Mol Genet 11: 1399–1407. 12023982 10.1093/hmg/11.12.1399

[2] Ma X, Liu L, Nie W, Li Y, Zhang B, Zhang J, et al (2013) Prognostic role of caveolin in breast cancer: a meta-analysis. Breast 22: 462–469. 10.1016/j.breast.2013.03.005 23639584

[3] Dai J, Tang K, Xiao W, Yu G, Zeng J, Li W, et al (2014) Prognostic significance of C-reactive protein in urological cancers: a systematic review and meta-analysis. Asian Pac J Cancer Prev 15: 3369–3375. 24870724 10.7314/apjcp.2014.15.8.3369

[4] Zeng R, Duan L, Kong Y, Liang Y, Wu X, Wei X, et al (2013) Clinicopathological and prognostic role of MMP-9 in esophageal squamous cell carcinoma: a meta-analysis. Chin J Cancer Res 25: 637–645. 10.3978/j.issn.1000-9604.2013.11.03 24385690 PMC3872548

[5] Yang H, Chen B (2013) CD147 in ovarian and other cancers. Int J Gynecol Cancer 23: 2–8. 10.1097/IGC.0b013e3182749139 23221648

[6] Liu Y, Tang W, Wang J, Xie L, Li T, He Y, et al (2013) Clinicopathological and prognostic significance of S100A4 overexpression in colorectal cancer: a meta-analysis. Diagn Pathol 8: 181 10.1186/1746-1596-8-181 24188373 PMC3833630

[7] Korpal M, Lee ES, Hu G, Kang Y (2008) The miR-200 family inhibits epithelial-mesenchymal transition and cancer cell migration by direct targeting of E-cadherin transcriptional repressors ZEB1 and ZEB2. J Biol Chem 283: 14910–14914. 10.1074/jbc.C800074200 18411277 PMC3258899

[8] Lu J, Getz G, Miska EA, Alvarez-Saavedra E, Lamb J, Peck D, et al (2005) MicroRNA expression profiles classify human cancers. Nature 435: 834–838. 15944708 10.1038/nature03702

[9] Gregory PA, Bert AG, Paterson EL, Barry SC, Tsykin A, Farshid G, et al (2008) The miR-200 family and miR-205 regulate epithelial to mesenchymal transition by targeting ZEB1 and SIP1. Nat Cell Biol 10: 593–601. 10.1038/ncb1722 18376396

[10] Hurteau GJ, Carlson JA, Roos E, Brock GJ (2009) Stable expression of miR-200c alone is sufficient to regulate TCF8 (ZEB1) and restore E-cadherin expression. Cell Cycle 8: 2064–2069. 19502803 10.4161/cc.8.13.8883

[11] Yang Y, Qian J, Chen Y, Pan Y (2014) Prognostic role of circulating microRNA-21 in cancers: evidence from a meta-analysis. Tumour Biol. 10.1007/s13277-014-1846-8 24664585

[12] Chen J, Zheng B, Wang C, Chen Y, Du C, Zhao G, et al. (2013) Prognostic role of microRNA-100 in various carcinomas: evidence from six studies. Tumour Biol. 10.1007/s13277-013-1398-3 24258109

[13] He J, Zhang F, Wu Y, Zhang W, Zhu X, He X, et al (2013) Prognostic role of microRNA-155 in various carcinomas: results from a meta-analysis. Dis Markers 34: 379–386. 10.3233/DMA-130984 23481631 PMC3810250

[14] Li M, Ma X, Li M, Zhang B, Huang J, Liu L, et al (2014) Prognostic Role of MicroRNA-210 in Various Carcinomas: A Systematic Review and Meta-Analysis. Dis Markers 2014: 106197 10.1155/2014/106197 24591754 PMC3925626

[15] Castilla MA, Diaz-Martin J, Sarrio D, Romero-Perez L, Lopez-Garcia MA, Vieites B, et al (2012) MicroRNA-200 family modulation in distinct breast cancer phenotypes. PLoS One 7: e47709 10.1371/journal.pone.0047709 23112837 PMC3480416

[16] Hur K, Toiyama Y, Takahashi M, Balaguer F, Nagasaka T, Koike J, et al (2013) MicroRNA-200c modulates epithelial-to-mesenchymal transition (EMT) in human colorectal cancer metastasis. Gut 62: 1315–1326. 10.1136/gutjnl-2011-301846 22735571 PMC3787864

[17] Leskela S, Leandro-Garcia LJ, Mendiola M, Barriuso J, Inglada-Perez L, Munoz I, et al (2011) The miR-200 family controls beta-tubulin III expression and is associated with paclitaxel-based treatment response and progression-free survival in ovarian cancer patients. Endocr Relat Cancer 18: 85–95. 10.1677/ERC-10-0148 21051560

[18] Marchini S, Cavalieri D, Fruscio R, Calura E, Garavaglia D, Nerini IF, et al (2011) Association between miR-200c and the survival of patients with stage I epithelial ovarian cancer: a retrospective study of two independent tumour tissue collections. Lancet Oncol 12: 273–285. 10.1016/S1470-2045(11)70012-2 21345725

[19] Tang H, Deng M, Tang Y, Xie X, Guo J, Kong Y, et al (2013) miR-200b and miR-200c as prognostic factors and mediators of gastric cancer cell progression. Clin Cancer Res 19: 5602–5612. 10.1158/1078-0432.CCR-13-1326 23995857

[20] Yu J, Ohuchida K, Mizumoto K, Sato N, Kayashima T, Fujita H, et al (2010) MicroRNA, hsa-miR-200c, is an independent prognostic factor in pancreatic cancer and its upregulation inhibits pancreatic cancer invasion but increases cell proliferation. Mol Cancer 9: 169 10.1186/1476-4598-9-169 20579395 PMC2909980

[21] Diaz T, Tejero R, Moreno I, Ferrer G, Cordeiro A, Artells R, et al. (2014) Role of miR-200 family members in survival of colorectal cancer patients treated with fluoropyrimidines. J Surg Oncol. 10.1002/jso.23572 24510588

[22] Song F, Yang D, Liu B, Guo Y, Zheng H, Li L, et al (2014) Integrated microRNA network analyses identify a poor-prognosis subtype of gastric cancer characterized by the miR-200 family. Clin Cancer Res 20: 878–889. 10.1158/1078-0432.CCR-13-1844 24352645

[23] Valladares-Ayerbes M, Reboredo M, Medina-Villaamil V, Iglesias-Diaz P, Lorenzo-Patino MJ, Haz M, et al (2012) Circulating miR-200c as a diagnostic and prognostic biomarker for gastric cancer. J Transl Med 10: 186 10.1186/1479-5876-10-186 22954417 PMC3494541

[24] Tanaka K, Miyata H, Yamasaki M, Sugimura K, Takahashi T, Kurokawa Y, et al (2013) Circulating miR-200c levels significantly predict response to chemotherapy and prognosis of patients undergoing neoadjuvant chemotherapy for esophageal cancer. Ann Surg Oncol 20 Suppl 3: S607–615. 10.1245/s10434-013-3093-4 23838916

[25] Madhavan D, Zucknick M, Wallwiener M, Cuk K, Modugno C, Scharpff M, et al (2012) Circulating miRNAs as surrogate markers for circulating tumor cells and prognostic markers in metastatic breast cancer. Clin Cancer Res 18: 5972–5982. 10.1158/1078-0432.CCR-12-1407 22952344

[26] Cao Q, Lu K, Dai S, Hu Y, Fan W (2014) Clinicopathological and prognostic implications of the miR-200 family in patients with epithelial ovarian cancer. Int J Clin Exp Pathol 7: 2392–2401. 24966949 PMC4069884

[27] Liu XG, Zhu WY, Huang YY, Ma LN, Zhou SQ, Wang YK, et al (2012) High expression of serum miR-21 and tumor miR-200c associated with poor prognosis in patients with lung cancer. Med Oncol 29: 618–626. 10.1007/s12032-011-9923-y 21516486

[28] Tejero R, Navarro A, Campayo M, Vinolas N, Marrades RM, Cordeiro A, et al (2014) miR-141 and miR-200c as markers of overall survival in early stage non-small cell lung cancer adenocarcinoma. PLoS One 9: e101899 10.1371/journal.pone.0101899 25003366 PMC4087018

[29] Toiyama Y, Hur K, Tanaka K, Inoue Y, Kusunoki M, Boland CR, et al (2014) Serum miR-200c is a novel prognostic and metastasis-predictive biomarker in patients with colorectal cancer. Ann Surg 259: 735–743. 10.1097/SLA.0b013e3182a6909d 23982750 PMC4032090

[30] Yu H, Duan B, Jiang L, Lin M, Sheng H, Huang J, et al (2013) Serum miR-200c and clinical outcome of patients with advanced esophageal squamous cancer receiving platinum-based chemotherapy. Am J Transl Res 6: 71–77. 24349623 PMC3853426

[31] Ceppi P, Mudduluru G, Kumarswamy R, Rapa I, Scagliotti GV, Papotti M, et al (2010) Loss of miR-200c expression induces an aggressive, invasive, and chemoresistant phenotype in non-small cell lung cancer. Mol Cancer Res 8: 1207–1216. 10.1158/1541-7786.MCR-10-0052 20696752

[32] Vilming Elgaaen B, Olstad OK, Haug KB, Brusletto B, Sandvik L, Staff AC, et al (2014) Global miRNA expression analysis of serous and clear cell ovarian carcinomas identifies differentially expressed miRNAs including miR-200c-3p as a prognostic marker. BMC Cancer 14: 80 10.1186/1471-2407-14-80 24512620 PMC3928323

[33] Hamano R, Miyata H, Yamasaki M, Kurokawa Y, Hara J, Moon JH, et al (2011) Overexpression of miR-200c induces chemoresistance in esophageal cancers mediated through activation of the Akt signaling pathway. Clin Cancer Res 17: 3029–3038. 10.1158/1078-0432.CCR-10-2532 21248297

[34] Kim MK, Jung SB, Kim JS, Roh MS, Lee JH, Lee EH, et al (2014) Expression of microRNA miR-126 and miR-200c is associated with prognosis in patients with non-small cell lung cancer. Virchows Arch 465: 463–471. 10.1007/s00428-014-1640-4 25124149

[35] Zhang GJ, Zhou T, Liu ZL, Tian HP, Xia SS (2013) Plasma miR-200c and miR-18a as potential biomarkers for the detection of colorectal carcinoma. Mol Clin Oncol 1: 379–384. 24649179 10.3892/mco.2013.61 PMC3915707

[36] Li J, Li X, Ren S, Chen X, Zhang Y, Zhou F, et al (2014) miR-200c overexpression is associated with better efficacy of EGFR-TKIs in non-small cell lung cancer patients with EGFR wild-type. Oncotarget 5: 7902–7916. 25277203 10.18632/oncotarget.2302 PMC4202169

[37] Tuomarila M, Luostari K, Soini Y, Kataja V, Kosma VM, Mannermaa A (2014) Overexpression of MicroRNA-200c Predicts Poor Outcome in Patients with PR-Negative Breast Cancer. PLoS One 9: e109508 10.1371/journal.pone.0109508 25329395 PMC4199599

[38] Wotschofsky Z, Busch J, Jung M, Kempkensteffen C, Weikert S, Schaser KD, et al (2013) Diagnostic and prognostic potential of differentially expressed miRNAs between metastatic and non-metastatic renal cell carcinoma at the time of nephrectomy. Clin Chim Acta 416: 5–10. 10.1016/j.cca.2012.11.010 23178446

[39] Gasparrini A, Armstrong B (2011) Multivariate meta-analysis: a method to summarize non-linear associations. Stat Med 30. 10.1002/sim.4226 PMC3841810 24293793

[40] Li J, Lenferink AE, Deng Y, Collins C, Cui Q, Purisima EO, et al (2010) Identification of high-quality cancer prognostic markers and metastasis network modules. Nat Commun 1: 34 10.1038/ncomms1033 20975711 PMC2972666

[41] Zhang X, Weng W, Xu W, Wang Y, Yu W, Tang X, et al (2014) Role of Bcl-2–938 C>A polymorphism in susceptibility and prognosis of cancer: a meta-analysis. Sci Rep 4: 7241 10.1038/srep07241 25430556 PMC5384243

[42] Wiklund ED, Bramsen JB, Hulf T, Dyrskjot L, Ramanathan R, Hansen TB, et al (2011) Coordinated epigenetic repression of the miR-200 family and miR-205 in invasive bladder cancer. Int J Cancer 128: 1327–1334. 10.1002/ijc.25461 20473948

[43] Lin CH, Jackson AL, Guo J, Linsley PS, Eisenman RN (2009) Myc-regulated microRNAs attenuate embryonic stem cell differentiation. EMBO J 28: 3157–3170. 10.1038/emboj.2009.254 19745813 PMC2744176

[44] Wiklund ED, Bramsen JB, Hulf T, Dyrskjot L, Ramanathan R, Hansen TB, et al (2011) miR-200c is upregulated by oxidative stress and induces endothelial cell apoptosis and senescence via ZEB1 inhibition. Cell Death Differ 18: 1628–1639. 10.1038/cdd.2011.42 21527937 PMC3172120

[45] Asaga S, Kuo C, Nguyen T, Terpenning M, Giuliano AE, Hoon DS (2011) Direct serum assay for microRNA-21 concentrations in early and advanced breast cancer. Clin Chem 57: 84–91. 10.1373/clinchem.2010.151845 21036945

[46] Komatsu S, Ichikawa D, Takeshita H, Tsujiura M, Morimura R, Nagata H, et al (2011) Circulating microRNAs in plasma of patients with oesophageal squamous cell carcinoma. Br J Cancer 105: 104–111. 10.1038/bjc.2011.198 21673684 PMC3137413

[47] Tomimaru Y, Eguchi H, Nagano H, Wada H, Kobayashi S, Marubashi S, et al (2012) Circulating microRNA-21 as a novel biomarker for hepatocellular carcinoma. J Hepatol 56: 167–175. 10.1016/j.jhep.2011.04.026 21749846

[48] Heegaard NH, Schetter AJ, Welsh JA, Yoneda M, Bowman ED, Harris CC (2012) Circulating micro-RNA expression profiles in early stage nonsmall cell lung cancer. Int J Cancer 130: 1378–1386. 10.1002/ijc.26153 21544802 PMC3259258

