# Supplementary material for: Prognostic Value and Clinicopathology Significance of MicroRNA-200c Expression in Cancer: A Meta-Analysis
Source: PLoS One. 2015 Jun 2;10(6):e0128642. doi: 10.1371/journal.pone.0128642 (PMC4452703; doi:10.1371/journal.pone.0128642)
Supplement: S1 Table — (DOC) [file pone.0128642.s011.doc]

**Table S1. Baseline characteristics of studies included in the meta-analysis.**

| **Study** | **Year** | **Country** | **Tumor type** | **Sample/male** | **Mean/Median Age** | **Tumor stage** | **Detection method** | **Cut-off for** | **High expression (%)** | **Sample type** | **Median follow-up date (month)** |
| --- | --- | --- | --- | --- | --- | --- | --- | --- | --- | --- | --- |
|  |  |  |  |  |  |  |  | **high expression** |  |  |  |
| Marchini | 2011 | Italy | ovarian cancer | 89/0 | NR | I | qRT-PCR | median | 72/144(50) | Tissue | NR |
| Tang | 2013 | China | gastric cancer | 126/70 | NR | I-IV | qRT-PCR | NR | 51/126 (39.8) | Tissue | NR |
| Yu | 2010 | Japan | pancreatic cancer | 99/62 | 65.7 | I-IV | qRT-PCR | 0.64 | NR | Tissue | 15 |
| Diaz | 2014 | Spain | colorectal cancer | 127/69 | 67.4 | I-III | qRT-PCR | -0.035 | 108/127 (85) | Tissue | NR |
| Song | 2014 | China | gastric cancer | 392/281 | 60.5 | I-IV | qRT-PCR | NR | 299(80.1) | Tissue | 35 |
| Ayerbes | 2012 | Spain | gastric cancer | 52/42 | NR | I-IV | qRT-PCR | median | NR | Blood | >48 |
| Tanaka | 2013 | Japan | esophageal cancer | 64/49 | NR | I-IV | qRT-PCR | median | 32(50) | Blood | NR |
| Madhavan | 2012 | Germany | breast cancer | 164/0 | NR | IV | qRT-PCR | 25th percentile as lower quartile | 123(75) | Blood | NR |
| Cao | 2014 | China | ovarian cancer | 100/0 | 58 | I-IV | qRT-PCR | median | 31(60) | Tissue | 36.8 |
| Liu | 2012 | China | lung cancer | 70/56 | 60 | I-IV | qRT-PCR | 2^△△CT >2.00 | NR | Tissue | 24 |
| Tejero | 2014 | Spain | lung cancer | 155/135 | 65 | I-III | qRT-PCR | defined by the Maxstat package of R | 108(70) | Blood | 43 |
| Yu | 2014 | China | esophageal cancer | 157/115 | 60 | III-IV | qRT-PCR | median | 78(50) | Blood | NR |
| Toiyama | 2013 | Japan | colorectal cancer | 182/105 | 68 | I-IV | qRT-PCR | median | 91(50) | Blood | NR |
| Ceppi | 2010 | Italy | lung cancer | 69/35 | NR | I-IV | qRT-PCR | median | 35(51) | Tissue | NR |
| Elgaaen | 2014 | Norway | ovarian cancer | 78/0 | 64 | I-IV | qRT-PCR | 33th percentile as higher quartile | 26(33) | Tissue | NR |
| Hamano | 2011 | Japan | esophageal cancer | 98/84 | NR | I-IV | qRT-PCR | median | 49(50) | Tissue | 28.8 |
| Kim | 2014 | Korea | lung cancer | 72/57 | 64 | I-IV | qRT-PCR | median | 36(50) | Tissue | 31 |
| Zhang | 2013 | China | colorectal cancer | 78/43 | 61 | I-IV | qRT-PCR | median | 39(50) | Tissue | NR |
| Li | 2014 | China | lung cancer | 150/84 | NR | III-IV | qRT-PCR | 2^-△CT≥0.01385 | 67(45) | Tissue | 16.7 |
| Tuomarila | 2014 | India | breast cancer | 172/0 | 60 | I-IV | qRT-PCR | median | 86(50) | Tissue | 116.4 |
| Leskelä | 2011 | Spain | ovarian cancer | 72/0 | 57 | I-IV | qRT-PCR | median | NR | Tissue | NR |
| Wotschofsky | 2013 | Germany | renal cell carcinoma | 89/64 | 65 | I-IV | qRT-PCR | NR | 45 (50.1) | Tissue | 33.8 |
| Torres | 2012 | Poland | endometrioid endometrial cancer | 122/0 | 62.8 | I-IV | qRT-PCR | median | 61(50) | Blood | NR |
