# Supplementary material for: Prognostic Value and Clinicopathology Significance of MicroRNA-200c Expression in Cancer: A Meta-Analysis
Source: PLoS One. 2015 Jun 2;10(6):e0128642. doi: 10.1371/journal.pone.0128642 (PMC4452703; doi:10.1371/journal.pone.0128642)
Supplement: S2 Table — (DOCX) [file pone.0128642.s012.docx]

**Table S2. The influence of individual study on the pooled estimate (OR) for overall survival**

| Study omitted | Year | HR | 95%CI | P value | Heterogeneity | |
| --- | --- | --- | --- | --- | --- | --- |
|  |  |  |  |  | I^2^ | P value |
| None |  | 1.41 | 0.95-2.10 | 0.09 | 81 | <0.00001 |
| Ayerbes | 2012 | 1.37 | 0.95-2.12 | 0.14 | 81 | <0.00001 |
| Cao | 2014 | 1.33 | 0.86-2.06 | 0.15 | 81 | <0.00001 |
| Diaz | 2014 | 1.52 | 0.96-2.41 | 0.05 | 81 | <0.00001 |
| Elgaaen | 2014 | 1.37 | 0.86-2.17 | 0.13 | 81 | <0.00001 |
| Kim | 2014 | 1.35 | 0.94-2.06 | 0.15 | 81 | <0.00001 |
| Li | 2014 | 1.52 | 0.96-2.42 | 0.05 | 80 | <0.00001 |
| Liu | 2012 | 1.33 | 0.85-2.04 | 0.16 | 80 | <0.00001 |
| Madhavan | 2012 | 1.34 | 0.94-2.08 | 0.15 | 81 | <0.00001 |
| Marchini | 2011 | 1.53 | 1.07-2.32 | 0.04 | 80 | <0.00001 |
| Song | 2014 | 1.43 | 0.97-2.26 | 0.11 | 81 | <0.00001 |
| Tanaka | 2013 | 1.46 | 0.98-2.18 | 0.11 | 81 | <0.00001 |
| Tang | 2013 | 1.53 | 1.07-2.31 | 0.04 | 78 | <0.00001 |
| Tejero | 2014 | 1.43 | 0.98-2.24 | 0.1 | 81 | <0.00001 |
| Toiyama | 2013 | 1.3 | 0.92-1.98 | 0.2 | 79 | <0.00001 |
| Torres | 2012 | 1.34 | 0.94-2.07 | 0.16 | 80 | <0.00001 |
| Yu | 2010 | 1.52 | 1.06-2.32 | 0.04 | 80 | <0.00001 |
| Yu | 2014 | 1.41 | 0.91-2.19 | 0.13 | 80 | <0.00001 |
