# Supplementary material for: Prognostic Value and Clinicopathology Significance of MicroRNA-200c Expression in Cancer: A Meta-Analysis
Source: PLoS One. 2015 Jun 2;10(6):e0128642. doi: 10.1371/journal.pone.0128642 (PMC4452703; doi:10.1371/journal.pone.0128642)
Supplement: S3 Table — (DOCX) [file pone.0128642.s013.docx]

**Table S3 The influence of individual study on the pooled estimate (OR) for progression-free survival**

| Study omitted | Year | HR | 95%CI | P value | Heterogeneity | |
| --- | --- | --- | --- | --- | --- | --- |
|  |  |  |  |  | I^2^ | P value |
| None |  | 1.12 | 0.68-1.84 | 0.67 | 80 | <0.0001 |
| Wotschofsky | 2013 | 1.09 | 0.64-1.87 | 0.74 | 83 | <0.00001 |
| Tanaka | 2013 | 1.08 | 0.63-1.86 | 0.78 | 83 | <0.00001 |
| Song | 2014 | 1.12 | 0.60-2.10 | 0.72 | 83 | <0.00001 |
| Marchini | 2011 | 1.25 | 0.74-2.09 | 0.4 | 80 | <0.0001 |
| Madhavan | 2012 | 0.95 | 0.61-1.48 | 0.83 | 61 | 0.02 |
| Li | 2014 | 1.3 | 0.82-2.06 | 0.27 | 67 | 0.006 |
| Leskelä | 2011 | 1.17 | 0.67-2.04 | 0.59 | 82 | <0.00001 |
| Ayerbes | 2012 | 1 | 0.58-1.73 | 0.99 | 81 | <0.0001 |

HR, hazard ratio; CI, confidence interval.
