# Supplementary material for: Prognostic Value and Clinicopathology Significance of MicroRNA-200c Expression in Cancer: A Meta-Analysis
Source: PLoS One. 2015 Jun 2;10(6):e0128642. doi: 10.1371/journal.pone.0128642 (PMC4452703; doi:10.1371/journal.pone.0128642)
Supplement: S4 Table — (DOCX) [file pone.0128642.s014.docx]

**Table S4 The influence of individual study on the pooled estimate (OR) for overall survival in Caucasians**

| Study omitted | Year | HR | 95%CI | P value | Heterogeneity | |
| --- | --- | --- | --- | --- | --- | --- |
|  |  |  |  |  | I^2^ | P value |
| None |  | 1.37 | 0.74-2.53 | 0.32 | 77 | 0.0002 |
| Ayerbes | 2012 | 1.25 | 0.61-2.55 | 0.54 | 79 | 0.0002 |
| Diaz | 2014 | 1.65 | 0.92-2.97 | 0.09 | 68 | 0.008 |
| Elgaaen | 2014 | 1.26 | 0.62-2.56 | 0.52 | 80 | 0.0002 |
| Madhavan | 2012 | 1.22 | 0.63-2.35 | 0.55 | 79 | 0.0002 |
| Marchini | 2011 | 1.68 | 0.97-2.93 | 0.06 | 70 | 0.007 |
| Tejero | 2014 | 1.38 | 0.64-2.95 | 0.41 | 81 | 0.0001 |
| Torres | 2012 | 1.2 | 0.61-2.36 | 0.6076 | 74 | 0.001 |

HR, hazard ratio; CI, confidence interval.
