# Supplementary material for: Prognostic Value and Clinicopathology Significance of MicroRNA-200c Expression in Cancer: A Meta-Analysis
Source: PLoS One. 2015 Jun 2;10(6):e0128642. doi: 10.1371/journal.pone.0128642 (PMC4452703; doi:10.1371/journal.pone.0128642)
Supplement: S5 Table — (DOCX) [file pone.0128642.s015.docx]

**Table S5 The influence of individual study on the pooled estimate (OR) for overall survival in Asians**

| Study omitted | Year | HR | 95%CI | P value | Heterogeneity | |
| --- | --- | --- | --- | --- | --- | --- |
|  |  |  |  |  | I^2^ | P value |
| None |  | 1.46 | 0.85-2.82 | 0.17 | 83 | <0.00001 |
| Cao | 2014 | 1.33 | 0.78-2.28 | 0.3 | 84 | <0.00001 |
| Kim | 2014 | 1.33 | 0.76-2.33 | 0.32 | 84 | <0.00001 |
| Li | 2014 | 1.69 | 0.96-3.06 | 0.09 | 82 | <0.00001 |
| Liu | 2012 | 1.31 | 0.76-2.26 | 0.34 | 84 | <0.00001 |
| Song | 2014 | 1.53 | 0.81-2.90 | 0.19 | 85 | <0.00001 |
| Tanaka | 2013 | 1.52 | 0.82-2.62 | 0.2 | 85 | <0.00001 |
| Tang | 2013 | 1.7 | 0.96-3.2 | 0.07 | 81 | <0.00001 |
| Toiyama | 2013 | 1.24 | 0.73-2.11 | 0.43 | 81 | <0.00001 |
| Yu | 2010 | 1.69 | 0.95-3.01 | 0.07 | 84 | <0.00001 |
| Yu | 2014 | 1.47 | 0.79-2.76 | 0.23 | 83 | <0.00001 |

HR, hazard ratio; CI, confidence interval.
