# Supplementary material for: Prognostic Value and Clinicopathology Significance of MicroRNA-200c Expression in Cancer: A Meta-Analysis
Source: PLoS One. 2015 Jun 2;10(6):e0128642. doi: 10.1371/journal.pone.0128642 (PMC4452703; doi:10.1371/journal.pone.0128642)
Supplement: S6 Table — (DOCX) [file pone.0128642.s016.docx]

**Table S6 The influence of individual study on the pooled estimate (OR) for overall survival in tissue samples**

| Study omitted | Year | HR | 95%CI | P value | Heterogeneity | |
| --- | --- | --- | --- | --- | --- | --- |
|  |  |  |  |  | I^2^ | P value |
| None |  | 0.99 | 0.59-1.67 | 0.97 | 79 | <0.00001 |
| Cao | 2014 | 0.89 | 0.54-1.48 | 0.67 | 79 | <0.00001 |
| Diaz | 2014 | 1.09 | 0.61-1.96 | 0.76 | 81 | <0.00001 |
| Elgaaen | 2014 | 0.89 | 0.52-1.53 | 0.69 | 78 | <0.00001 |
| Kim | 2014 | 0.86 | 0.52-1.45 | 0.58 | 78 | <0.00001 |
| Li | 2014 | 1.11 | 0.60-2.06 | 0.74 | 80 | <0.00001 |
| Liu | 2012 | 0.86 | 0.52-1.43 | 0.56 | 78 | <0.0001 |
| Marchini | 2011 | 1.12 | 0.65-1.92 | 0.68 | 80 | <0.00001 |
| Song | 2014 | 0.96 | 0.54-1.73 | 0.9 | 78 | <0.0001 |
| Tang | 2013 | 1.12 | 0.62-2.02 | 0.71 | 79 | <0.00001 |
| Yu | 2010 | 1.11 | 0.63-1.96 | 0.73 | 80 | <0.00001 |

HR, hazard ratio; CI, confidence interval.
