# Supplementary material for: Prognostic Value and Clinicopathology Significance of MicroRNA-200c Expression in Cancer: A Meta-Analysis
Source: PLoS One. 2015 Jun 2;10(6):e0128642. doi: 10.1371/journal.pone.0128642 (PMC4452703; doi:10.1371/journal.pone.0128642)
Supplement: S7 Table — (DOCX) [file pone.0128642.s017.docx]

**Table S7 The influence of individual study on the pooled estimate (OR) for overall survival in blood samples**

| Study omitted | Year | HR | 95%CI | P value | Heterogeneity | |
| --- | --- | --- | --- | --- | --- | --- |
|  |  |  |  |  | I^2^ | P value |
| None |  | 2.1 | 1.52-2.90 | <0.00001 | 32 | 0.19 |
| Ayerbes | 2012 | 2.11 | 1.44-3.10 | 0.0001 | 42 | 0.12 |
| Madhavan | 2012 | 2.04 | 1.45-2.87 | <0.0001 | 37 | 0.16 |
| Tanaka | 2013 | 2.18 | 1.52-3.11 | <0.0001 | 42 | 0.13 |
| Tejero | 2014 | 2.3 | 1.65-3.19 | <0.00001 | 21 | 0.27 |
| Toiyama | 2013 | 1.85 | 1.43-2.39 | <0.00001 | 0 | 0.46 |
| Torres | 2012 | 2 | 1.38-2.90 | 0.0002 | 34 | 0.18 |
| Yu | 2014 | 2.31 | 1.55-3.46 | <0.0001 | 33 | 0.19 |

HR, hazard ratio; CI, confidence interval.
